# Oncology and medical education—past, present and future

**DOI:** 10.3332/ecancer.2016.ed54

**Published:** 2016-05-16

**Authors:** Judith Cave

**Affiliations:** Dept of Medical Oncology, University Hospital Southampton NHS Foundation Trust, Tremona Road, Southampton, SO16 6YD, UK

**Keywords:** oncology, medical education

## Abstract

Oncologists should contribute to the undergraduate curriculum whenever they can, and should teach communication skills, acute oncology, prescribing, and other transferable skills. Newly qualified doctors will care for many patients with cancer in their first years of work, and all doctors need to know when an urgent oncology referral is required and to be aware of the pace of change in oncology. Oncologists should involve their patients in teaching whenever it is appropriate. We should aim to inspire junior doctors to consider a career in oncology. The oncology education community should adopt new teaching methods, for example simulation, mock MDTs and student led clinics. CPD provided by honorable organisations, including online learning, is becoming more important for oncologists to keep up to date.

## Introduction

Recent political and scientific developments have brought new challenges for medical educators in general, and oncologists in particular. Some of the challenges are born out of success, for example those accompanied by the invention of a new class of immune checkpoint blockade drugs. Some of the challenges are a consequence of increasing the professionalisation of medical education and increasing the regulation of funding. There are also challenges associated with the current learning and working environment: in the UK, the National Health Service is under pressure to find efficiency savings, junior doctors are currently undertaking industrial action, and there is the possibility of a shortage of junior doctors on the horizon. So how can oncologists rise to these challenges?

## Undergraduate teaching and learning

Oncology is a relatively new discipline, and there is sometimes a tendency for it to be regarded as a postgraduate specialty. A survey of medical schools in 2011 found that only 36% of medical schools included a dedicated period of time for oncology learning in their curriculum [1]. The median length of time was 2 weeks (range 2–3). A survey of newly qualified doctors performed in 2005 found that 31% recalled meeting fewer than 10 patients with cancer at medical school [2]. One out of every 2 people born after 1960 in the UK will be diagnosed with some form of cancer during their lifetime [3], so it seems surprising that there have historically been such low levels of undergraduate exposure to oncology and patients with cancer. Should we be asking our medical schools to justify this or to change it? Perhaps not. It is important to remember that it is not only oncologists who teach about cancer. In fact most disciplines will include reference to malignancy in their undergraduate teaching. So a more important question is: what can oncologists most helpfully contribute to the undergraduate curriculum?

Newly qualified doctors will look after many inpatients with cancer, and the most complex cases will require long hospital stays. Newly qualified doctors therefore need the skills to care for and communicate with these patients and their families. The GMC in its 2014 document on the state of undergraduate training recognized that communication skills are still an area where new doctors feel underprepared [4]. Oncologists are ideally situated to role model and teach honest and compassionate communication. New doctors also need an awareness of oncological emergencies so they can appropriately triage patients to the acute oncology service. We must teach about neutropenic sepsis and cord compression at medical school.

Going to medical school isn’t just about preparing to be a Foundation doctor. Oncologists can teach the knowledge, skills and attitudes that doctors will need throughout their career, including an awareness of the pace of scientific change, knowledge about how to access information about unfamiliar compounds and their toxicities, excellence in prescribing drugs with a narrow therapeutic window, and planning and managing the transition to end of life care. Oncologists can perhaps most helpfully make sure that medical students meet patients with cancer. There is evidence that meeting more patients with cancer at medical school is correlated with preparedness for practice [2]. Students given the opportunity to care for patients in a hospice reported learning sensitivity to human suffering, and compassion [5]. Student-run clinics are always enjoyed and valued by students [6] and the relatively protocol-driven nature of some elements of cancer care could lend themselves well to a student-run clinic. Any such novel approach would of course need to be carefully supervised and evaluated. Many of our patients however will feel passionately that they wish to be involved in educating the next generation of doctors, and will be uniquely equipped to do so.

As well as involving patients in teaching, oncologists should involve the entire multi-professional team. Simulation can be a successful method, but it has not been widely adopted by oncologists as an educational tool. There are experiences, such as participating in multidisciplinary team meetings, which lend themselves well to a simulation based approach. This is something which should be explored and evaluated and published if successful so that a body of literature on teaching about cancer can be built up and best practice can be shared.

## Postgraduate education for non-oncologists

Postgraduate medical education in the UK is experiencing a difficult time, with current industrial action at the forefront of everyone’s mind and the time and financial pressures on doctors greater than ever. Specialties are likely to be competing for trainees, and so the onus is on us as a profession to ensure that working on the oncology wards is highly educational. We need to teach junior doctors on every ward round and in the clinic whenever we can. Most oncologists are expert communicators, and medicine is a cognitive apprenticeship. We can provide opportunities for junior staff to learn even by simple changes such as getting into the habit of verbalising our thought processes and decision making before, during and after difficult consultations.

Given the scientific opportunities and the pace of development of new cancer drugs it should not be difficult to inspire young doctors about oncology, but we need to make sure information is presented in an accessible fashion. Most oncology departments run journal clubs and seminars, and all members of staff must be welcomed. Some specialties are running events to educate and enthuse junior doctors, for example the popular ‘Geriatrics for Juniors’ conference (aeme.org.uk/g4j/). There is currently no junior doctors’ cancer care forum in the UK but perhaps one should be developed. Other specialties have successfully used social media to disseminate information presented at conferences more widely, and specialties such as emergency medicine have led the way (#EMConf). Whilst oncology conferences now usually have twitter feeds, these are not currently aimed at trainees or non-oncologists and this is something which could be improved.

## Postgraduate oncology education and continuing professional development (CPD)

There is a pressing need for oncologists to keep up to date. The FDA granted 31 approvals for new oncology drugs or new indications for drugs in 2015 alone [7]. In the past, international conferences have been an excellent source of CPD for oncologists. The pharmaceutical industry has contributed substantially to the cost of CPD both by funding conferences and by supporting individuals to attend. Increased regulation and transparency is now demanded by the Association of British Pharmaceutical Industry (ABPI) code of practice. From 2015 companies have been required to declare how much sponsorship has been given to healthcare professionals and to name the recipients. It seems likely that this will have a huge impact on pharmaceutical funding of CPD, and some would argue that this is for the best. We need to work together with colleagues in industry to find a way to continue the strong tradition of science and education in oncology without compromising the needs of either party.

The traditional ‘bums on seats’ model of CPD is expensive but also not very educationally sound. Adults learn best when they are engaged and participating. Canadian physicians who participate in CPD are less likely to receive patient complaints, and the greatest effect is from group-based CPD (compared to self-directed learning or assessment based activities) [8]. Postgraduate oncology education could benefit from being more focused on case discussions and small group learning. Formalised processes such as action learning sets or mock MDT meetings could be used to facilitate this.

Technology enhanced learning is undergoing massive expansion because of increasing access to the necessary hardware and software. There are a huge number of different forms that technology enhanced learning can take, but the majority can now be accessed from a mobile device which is attractive for busy clinicians. Unfortunately, because research has not kept up with the technological advances, it is very difficult to know how effective technology enhanced learning methods are. It can be said that online platforms including *e*cancer.org are a democratic and open route to high quality CPD. Online CPD can also be used in a very learner focused and targeted way, so that oncologists can access what they need, when they have time, with no barriers. Appraisal practice and documentation needs to recognize and validate online learning.

Donors who are able to support oncology CPD could consider weighting donations towards honorable organisations instead of individual clinicians. Bodies such as the Union for International Cancer Control (UICC) produce high quality educational materials and online resources. These organisations are very patient focused, and their involvement should achieve the joint aims of impartiality and a strong basis in clinical practice.

## Conclusions

Oncologists need to seize any opportunity to be involved in undergraduate medical education, and when we do get involved, we should teach transferable skills. We should ensure that junior doctors working in oncology posts gain the maximum educational value from this. We should strive to increase the educational value of the hours we spend keeping up to date, and consider learning with colleagues in small groups and utilising on-line CPD platforms.
